# Antibiotic Resistance and Plasmid Profile Analysis of *Salmonella* Enteritidis Isolated in Siberia and the Far East of Russia between 1990 and 2017

**DOI:** 10.3390/pathogens10101240

**Published:** 2021-09-25

**Authors:** Alexey V. Rakov, Natalya A. Kuznetsova

**Affiliations:** Laboratory of Molecular Epidemiology and Ecology of Pathogenic Bacteria, Somov Institute of Epidemiology and Microbiology, 690087 Vladivostok, Russia; kuznetsovanata@mail.ru

**Keywords:** *Salmonella*, *Salmonella* Enteritidis, bacterial pathogens, antibiotics, resistance, plasmid profiles, Russia

## Abstract

*Salmonella* is one of the major causes of foodborne disease outbreaks globally. Specifically, *Salmonella enterica* subsp. *enterica* serovar Enteritidis (*S.* Enteritidis) is one of the major causes of zoonotic *Salmonella* infection in humans worldwide. In this study, we present data on antimicrobial resistance (AMR) and plasmid profiles of *S.* Enteritidis strains isolated from patients, food, and the environment in Siberia and the Far East of Russia obtained during *Salmonella* monitoring between 1990 and 2017. A total of 345 *S.* Enteritidis isolates were tested by the disk diffusion method with a set of 15 antibiotics using EUCAST breakpoints v. 10 and by plasmid profile analysis using the alkaline lysis method. The results have shown a substantial decrease in susceptibility to aminoglycosides and quinolones during the study period. No significant differences were found in the susceptibility of strains between regions as well as in the its correlation with different plasmid types of the pathogen. Several *S.* Enteritidis strains were found to be resistant to ampicillin, kanamycin, tetracycline, chloramphenicol, and cephalosporins. All tested *S.* Enteritidis strains were susceptible only to imipenem. In this study, we observed a relatively low level of AMR in *S.* Enteritidis strains isolated in Siberia and the Far East of Russia. Nevertheless, it is important to continue the molecular genetic monitoring and AMR surveillance of *S.* Enteritidis to track further increases in AMR using conventional phenotypic susceptibility testing and by introducing whole-genome sequencing to identify AMR mechanisms.

## 1. Introduction

Salmonellosis remains one of the most concerning bacterial enteric infections throughout Russia. This is confirmed by morbidity rates over the last five years, both in the country and at the regional levels. According to official statistics, in 2018, the average morbidity rate for salmonellosis in Russia was 22.9 per 100,000 population, while in at least 17 territories, rates exceeded the national average [1].

Patients with *Salmonella* infection are commonly not treated with antimicrobial drugs. Nevertheless, the effectiveness of their use depends directly on the susceptibility of the pathogen to them. In Russia, most *Salmonella* strains identified in microbiological laboratories of medical institutions are not tested for susceptibility to antibiotics. This is because current standards do not regulate the antibiogram guidelines for salmonellosis, and empiric therapy is commonly used. An analysis of the available literature, however, shows an increase in the drug resistance of *Salmonella* strains to antimicrobial agents in different regions of Russia and worldwide due to misuse [2,3]. Increasing antimicrobial resistance (AMR) and the emergence of multiple drug resistant (MDR) *Salmonella* strains have increased the need to understand the susceptibility of *Salmonella* strains that are currently circulating in different countries and their administrative regions to antimicrobial drugs for adequate etiotropic therapy [4,5].

Our previous studies have shown that the dissemination of some *Salmonella* populations is more influenced by globalization processes, while other populations of the pathogen are circulated in local poultry farms for years [6]. We assumed that these features could affect the antibiotic resistance spectrum of *Salmonella* populations circulating in the administrative territories of Siberia and the Far East of Russia. Since AMR strains are one of the most dangerous threats to public health, the antibiotic resistance monitoring of *Salmonella* is an important element of the surveillance system. An important component of surveillance is the integrated use of methods for studying both antibiotic susceptibility and genetic characteristics of the pathogen.

*Salmonella enterica* subsp. *enterica* serovar Enteritidis (*S.* Enteritidis) is one of the most frequent serovars of zoonotic *Salmonella* infection in humans worldwide [7], including Russia [1,6,8]. The aim of the study was to compare the antibiotic susceptibility of *S.* Enteritidis populations that were isolated in different regions of Siberia and the Russian Far East between 1990 and 2017 based on geography, period of isolation, and the plasmid content.

## 2. Results

**Figure 1:**
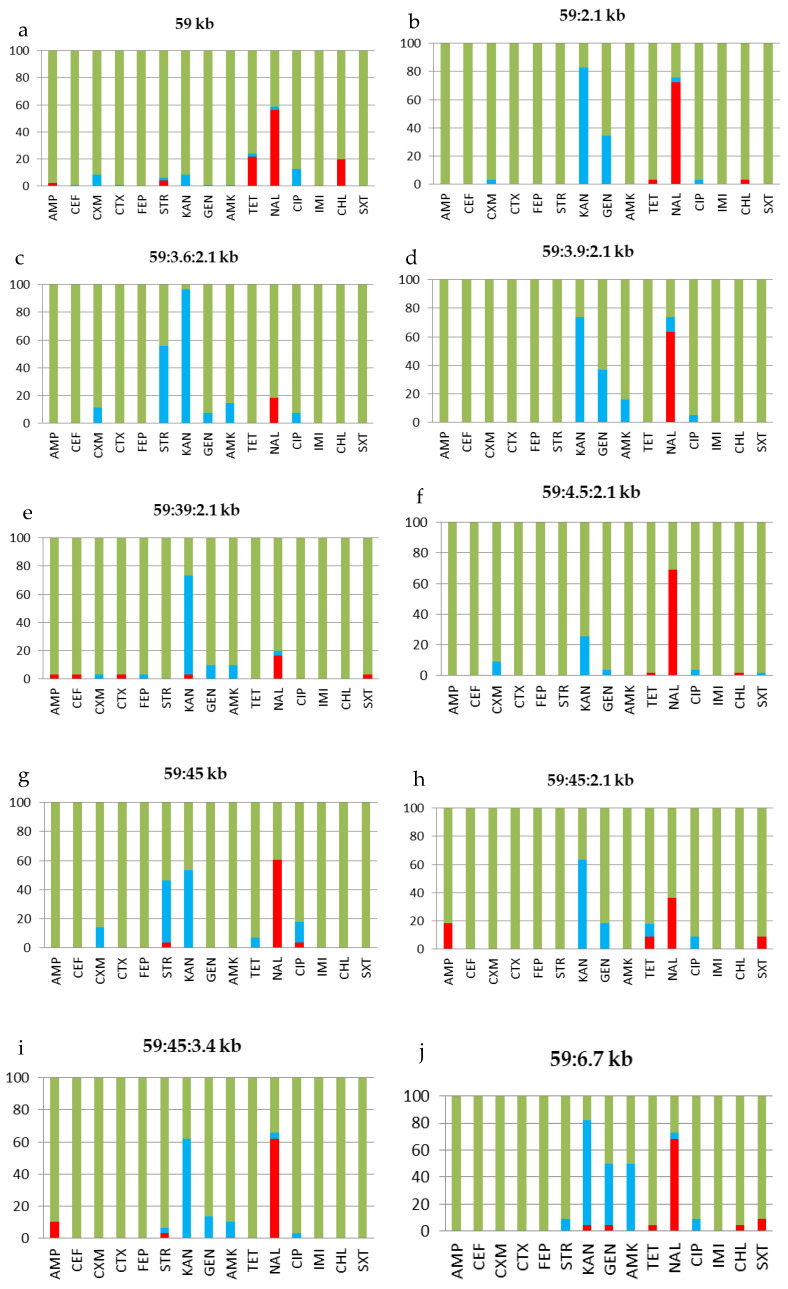
Antibiotic susceptibility of *S.* Enteritidis strains (n=345) of the top ten plasmid profile types isolated in regions of Siberia and the Far East of Russia: (**a**) 59 kb, (**b**) 59:2.1 kb, (**c**) 59:3.6:2.1 kb, (**d**) 59:3.9:2.1 kb, (**e**) 59:39:2.1 kb, (**f**) 59:4.5:2.1kb, (**g**) 59:45 kb, (**h**) 59:45:2.1 kb, (**i**) 59:45:3.4 kb, and (**j**) 59:6.7 kb. The Y-axis indicates the percentage of strains susceptible (green), resistant (red), and intermediate (blue) to the given antibiotic. Ampicillin 10 μg (AMP), cephalexin 30 μg (CEF), cefuroxime 30 μg (CXM), cefotaxime 30 μg (CTX), cefepime 30 μg (FEP), streptomycin 10 μg (STR), kanamycin 30 μg (KAN), gentamicin 30 μg (GEN), amikacin 30 μg (AMK), tetracycline 30 μg (TET), nalidixic acid 30 μg (NAL), ciprofloxacin 5 μg (CIP), imipenem 10 μg (IMI), chloramphenicol 30 μg (CHL), and trimethoprim 1.25 μg/sulfamethoxazole 23.75 μg (SXT).

All tested *S.* Enteritidis strains belonged to the top ten plasmid types of the pathogen found to be widespread in the abovementioned regions of Russia: 59 kb (96 strains), 59:2.1 kb (29 strains), 59:3.6:2.1 kb (27 strains), 59:3.9:2.1 kb (19 strains), 59:39:2.1 kb (30 strains), 59:4.5:2.1kb (55 strains), 59:45 kb (28 strains), 59:45:2.1 kb (11 strains), 59:45:3.4 kb (28 strains), and 59:6.7 kb (22 strains) [6]. The antibiotic susceptibility patterns of *S.* Enteritidis strains were studied in the context of the plasmid profile. This profile allowed for each of the ten major plasmid types (profiles) of the pathogen to be presented, which were identified in almost all regions of Siberia and the Far East of Russia included in the study. Table 1 and Figure 1 show antibiotic resistance patterns for each of the top ten plasmid types of *S.* Enteritidis circulating in Siberia and the Far East of Russia. The results showed that the percentage of susceptible (S), resistant (R), and intermediate (I) strains for each plasmid type is different. This is consistent with the antibiotic susceptibility of all *S.* Enteritidis strains circulating in Siberia as well as in the southern and northern territories of the Far East of Russia. Thus, for different plasmid types of the pathogen, the antibiotic resistance pattern could also be slightly different. These differences may not depend on a specific plasmid profile but on a period of time (i.e., date of sampling) or a specific region. Next, we split the strains into groups by date and location.

**Figure 2:**
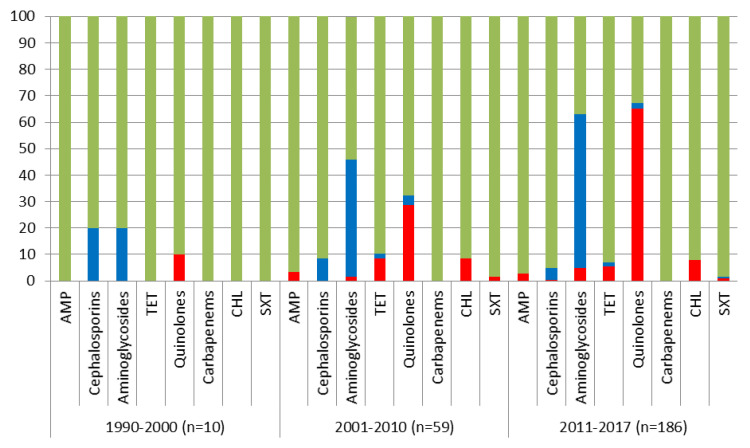
The change in antibiotic susceptibility of *S.* Enteritidis strains during the three periods (period I (1990–2000), period II (2001–2010), and period III (2011–2017)). The Y-axis indicates the percentage of strains susceptible (green), resistant (red), and intermediate (blue) to the given antibiotic. Ampicillin 10 μg (AMP), tetracycline 30 μg (TET), chloramphenicol 30 μg (CHL), and trimethoprim 1.25 μg/sulfamethoxazole 23.75 μg (SXT).

To assess the change in the susceptibility of *S.* Enteritidis strains over time, they were split into three periods according to decade (period I (1990–2000), period II (2001–2010), and period III (2011–2017)). As can be seen in Figure 2, in period III, there was a significant decrease in the number of susceptible strains to the aminoglycosides (period II: 54.2%±6.5% vs. period III: 37.1 ± 3.5%, *p* = 0.021) and a decrease in the percentage of susceptible strains to the quinolones (67.8 ± 6.1% vs. 33.9 ± 3.5%, *p* = 0.000002, respectively). Furthermore, only the quinolones showed a significant increase in resistant strains in period III (28.8 ± 5.9% vs. 65.0 ± 3.5%, *p* = 0).

**Figure 3:**
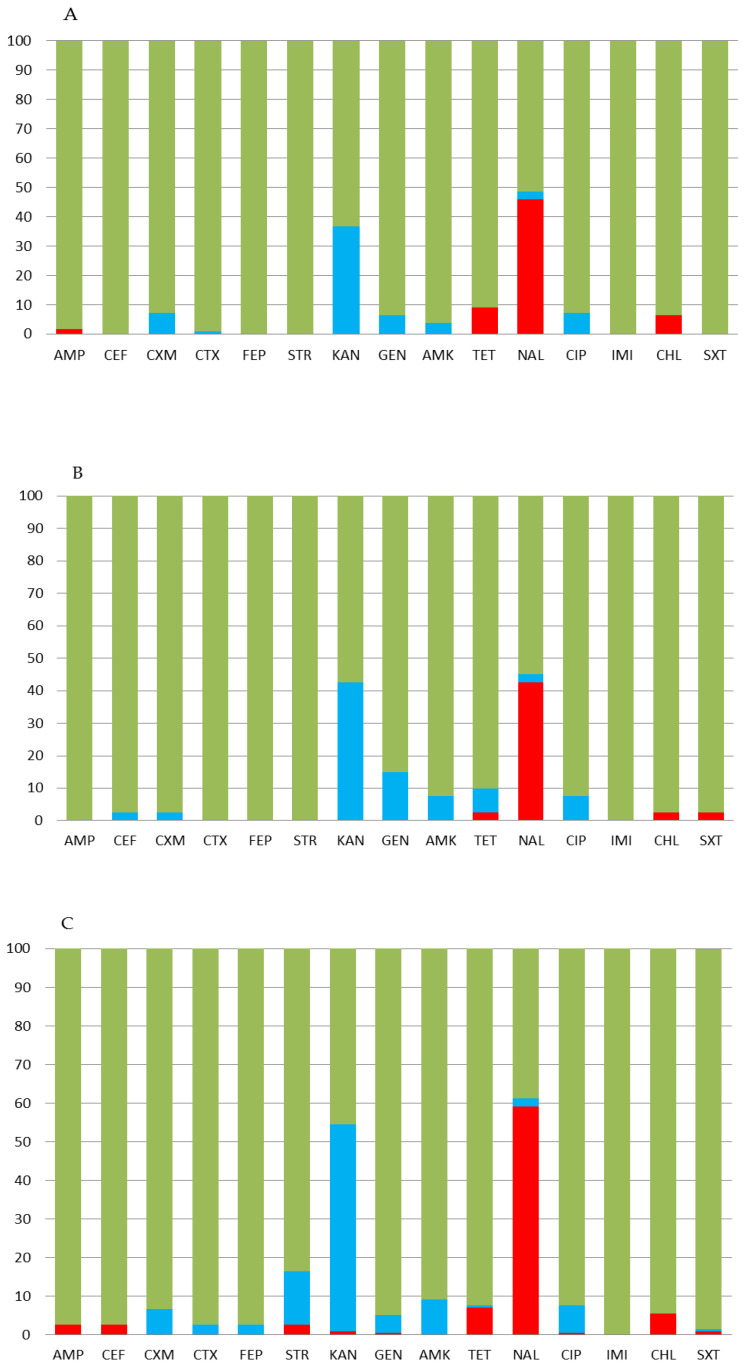
Antibiotic susceptibility of *S.* Enteritidis strains of the top ten plasmid profile types isolated in regions of (**A**) Siberia, and (**B**) the northern and (**C**) southern Far East of Russia. The Y-axis indicates the percentage of strains susceptible (green), resistant (red), and intermediate (blue) to the given antibiotic. Ampicillin 10 μg (AMP), cephalexin 30 μg (CEF), cefuroxime 30 μg (CXM), cefotaxime 30 μg (CTX), cefepime 30 μg (FEP), streptomycin 10 μg (STR), kanamycin 30 μg (KAN), gentamicin 30 μg (GEN), amikacin 30 μg (AMK), tetracycline 30 μg (TET), nalidixic acid 30 μg (NAL), ciprofloxacin 5 μg (CIP), imipenem 10 μg (IMI), chloramphenicol 30 μg (CHL), and trimethoprim 1.25 μg/sulfamethoxazole 23.75 μg (SXT).

Thus, long-term monitoring revealed a significant decrease in the susceptibility of the pathogen to aminoglycosides and quinolones in the 2010s. Moreover, in the last period of surveillance, strains resistant to ampicillin, kanamycin, tetracycline, chloramphenicol, and even cephalosporins emerged. The distribution of the strains sensitive to antibiotics in each of the three abovementioned regions is shown in Figure 3. Regardless of the region studied and the plasmid type of circulating strains, 100% susceptibility of the pathogen only to imipenem was revealed. The maximum percentage of resistant strains was found in the quinolones: 42.5 ± 7.8% in the northern territories of the Far East, 46.8 ± 4.4% in Siberia, and 59.2 ± 3.6% in the southern territories of the Far East of Russia (nonsignificant differences, *p* = 0.0525). In addition, in all regions, *S.* Enteritidis strains largely showed intermediate resistance to the aminoglycosides: 36.7 ± 4.6% of the tested strains in Siberia, 42.5 ± 7.8% in the northern Far East, and 53.1 ± 3.6% in the southern Far East. It was revealed that *S.* Enteritidis strains with intermediate resistance to aminoglycosides are significantly more common in the south of the Far East than in Siberia (*p* = 0.0052).

The antibiotic susceptibility of *S.* Enteritidis strains isolated from various environmental sources in the Far East and Siberia of Russia is shown in Table 2. When analyzing the results of the strains isolated from sporadic cases, it was found that the percentage of susceptible strains to antibiotics of different groups ranged from 44.3 ± 3.1% for nalidixic acid to 100% for imipenem. Intermediate resistant strains were found from 0.4 ± 0.4% for cephalosporins to 51.8 ± 3.1% for kanamycin. Among sporadic cases, the highest percentage of resistant strains was found for nalidixic acid: 53.7 ± 3.1%. When studying strains isolated from patients during outbreaks, the susceptibility to nalidixic acid was the least, 36.6 ± 7.5% of strains; intermediate resistance was found to different groups of antibiotics; and their percentage ranged from 2.4 ± 2.4% to 34.1 ± 7.4%. Resistance to nalidixic acid in this group of strains was also high, 61.0 ± 7.6%. In strains isolated from food, the same pattern appeared: the smallest number of susceptible strains was found for nalidixic acid, 43.5 ± 7.3%; intermediate resistant strains were up to 32.6 ± 6.9% (to kanamycin); and resistance to nalidixic acid was the highest, 52.2 ± 7.4%. The number of environmental samples (Table 2) were too few for statistical inference (n = 3). Despite this, strains with intermediate resistance to kanamycin and resistant to nalidixic acid were identified.

The results of the antibiotic susceptibility testing of *S.* Enteritidis strains isolated regardless of the epidemiological status (sporadic or outbreak) and the ecological niche (patient, food, or the environment) showed that, in general, the pathogen is most resistant to nalidixic acid. All tested *S.* Enteritidis show susceptibility to imipenem.

## 3. Discussion

Long-term monitoring showed that the *S.* Enteritidis population in Siberia and the Far East of Russia is highly heterogeneous but mostly limited to the top ten plasmid types of the pathogen: 59 kb, 59:2.1 kb, 59:3.6:2.1 kb, 59:3.9:2.1 kb, 59:39:2.1 kb, 59:4.5:2.1kb, 59:45 kb, 59:45:2.1 kb, 59:45:3.4 kb, and 59:6.7 kb [6]. It is important to emphasize that these ten plasmid types have been identified in almost all studied regions of Siberia and the Far East of Russia. These plasmids are mostly cryptic and do not carry antimicrobial resistance genes [6]. The first thing that should be noted is that, regardless of the plasmid profile, the susceptibility to antibiotics in the pathogen is mainly developed strictly within the pattern we have identified. In particular, the pathogen marked with the single 59 kb virulence plasmid showed resistance to ampicillin, streptomycin, tetracycline, chloramphenicol, and especially nalidixic acid. This plasmid has a ubiquitous distribution and was detected in the first years of monitoring. Resistance to six antibiotics, including kanamycin, gentamicin, and trimethoprim/sulfamethoxazole was identified among the strains of plasmid type 59:6.7 kb (Table 1). Figure 1 also depicts the antibiogram of the *S.* Enteritidis, the plasmid types of which became widespread throughout the regions in a later period. The strains with plasmid type 57:3.9:2.1 kb were responsible for the emergence of cephalosporins resistance.

The resistant *S.* Enteritidis strains have a specific regional distribution with strains resistant to tetracycline, chloramphenicol, and nalidixic acid in the western territories of Siberia having been identified (Figure 2). We observed the same pattern in the northern territories of the Far East of Russia. At the same time, findings in the Krasnoyarsk Oblast, which was not included in our study, showed a significant proportion of studied *S.* Enteritidis strains resistant to tetracycline, and only 26.2%±8.3% were susceptible to the drug [9]. In addition, in the Perm Krai, only 68.3% of the studied *Salmonella* strains were susceptible to nalidixic acid. Given the lowest antibacterial activity of drugs such as doxycycline and nitrofurantoin (sensitive to 31.8% and 30.0%, respectively) to regional *Salmonella* strains, researchers drew the attention of clinicians to the futility of their active use [10]. In our study, the number of antibiotics to which resistant *Salmonella* strains were found turned out to be greater in the southern territories of the Far East of Russia than in the northern territories or Siberia. Interestingly, a strain with resistance to cephalosporins was also identified there.

Nevertheless, as our studies have shown, the development of AMR in microorganisms is not the result of direct adaptation to the area of their circulation and the frequency of antibiotics use. Nalidixic acid has not been used for a long time either in medicine or in veterinary medicine, but resistance to it is increasing. On the contrary, gentamycin is not widely used as a first line antibiotic due to its toxic effects, while the microbe has developed a relatively low resistance to it.

Despite some differences, the overwhelming majority of *S.* Enteritidis strains, regardless of the plasmid profile and the region of isolation, represent a relatively homogeneous group in terms of antibiotic susceptibility. The differences in antibiotic susceptibility that we identified were noted in certain plasmid types, which so far has played a very limited role in the epidemiology of salmonellosis in Russia. Therefore, the process of the development of AMR in *S.* Enteritidis is mainly influenced by factors shared by different regions. In terms of the treatment of *Salmonella* infection, monitoring of the antibiotic resistance in *Salmonella* in a particular region is not as important as it might have been, given the size of Russia and its territories. Therefore, further studies of the mechanisms of antibiotic resistance development are necessary. In this regard, reasons for the development of AMR and the possible consequences of their use in the future remains highly important [4].

To our knowledge, this is the first study utilizing a combination of plasmid profiling and antimicrobial susceptibility testing of *S.* Enteritidis strains isolated between 1990 and 2017 from Russia. This work made it possible to learn more about the current state of AMR and the plasmid profiles of *S.* Enteritidis in poorly studied regions of Siberia and the Far East of Russia. Nevertheless, it is important to continue the molecular genetic monitoring and AMR surveillance of *S.* Enteritidis to track further increases in AMR using conventional phenotypic susceptibility testing and by introducing whole-genome sequencing to identify AMR mechanisms.

## 4. Materials and Methods

Long-term microbiological monitoring of *Salmonella* allowed us to maintain a collection of strains isolated in the last 30 years in Siberia and the Far East of Russia [6]. All *Salmonella* strains were stored at –80 °C. From this collection, a total of 345 *S.* Enteritidis strains (about 10% of the collection) isolated from different ecological niches and with different epidemiological backgrounds during the period 1990–2017 were studied. In particular, 255 strains were isolated as sporadic cases, 41 strains were outbreak strains, 46 strains were recovered from food, and 3 strains were from environmental samples. Monitoring of the *Salmonella* populations included the study of isolates from Siberia (Republic of Buryatia, Novosibirsk, Omsk, Tomsk, and Irkutsk Oblasts), and the northern (Kamchatka Krai, Magadan Oblast, Chukotka Autonomous Okrug, and the Republic of Sakha (Yakutia)) and southern regions of the Far East (Jewish Autonomous Okrug, Sakhalin Oblast, Khabarovsk, and Primorsky Krais) of Russia.

*Salmonella* serotyping was performed using the White–Kauffman–Le Minor scheme. Plasmid profile analysis was performed using the alkaline lysis method [11]. Then, plasmid DNA was electrophoresed in 0.7% agarose gel in 1× TBE buffer at 120 V for 2.5 h to estimate molecular weight. Plasmid sizes were determined with known *E. coli* plasmids: pBR322 (4.3 kb), pCT105 (7.5 kb), and RP4 (56 kb). The plasmid type (profile) was designated as the molecular weight of discovered plasmids in kilobases (kb) from large to small separated by a colon, similar to [12]. The susceptibility of *S.* Enteritidis strains to antibiotics was determined by the Kirby–Bauer disk diffusion test on Mueller–Hinton agar (Condalab, Madrid, Spain), including ampicillin 10 μg (AMP), cephalexin 30 μg (CEF), cefuroxime 30 μg (CXM), cefotaxime 30 μg (CTX), cefepime 30 μg (FEP), streptomycin 10 μg (STR), kanamycin 30 μg (KAN), gentamicin 30 μg (GEN), amikacin 30 μg (AMK), tetracycline 30 μg (TET), nalidixic acid 30 μg (NAL), ciprofloxacin 5 μg (CIP), imipenem 10 μg (IMI), chloramphenicol 30 μg (CHL), and trimethoprim 1.25 μg/sulfamethoxazole 23.75 μg (SXT). The *Escherichia coli* strains ATCC 25922 and ATCC 35218 were used as quality control strains according to the rules of the Clinical and Laboratory Standards Institute (CLSI) [13]. The antibiotic zone diameter breakpoints were assigned according to the EUCAST v. 10 guidelines [14]. The results obtained were processed by statistical methods using Student’s *t*-test and significance level (*p*-value) in Prism v. 7.0 (GraphPad Software, San Diego, CA, USA).

## Figures and Tables

**Table 1:** Resistance patterns for the ten most frequent plasmid profile types of *S.* Enteritidis strains (n = 345).

Plasmid Type (kb)	Number of Strains (%) with Resistance Pattern													
	Susceptible	AMP CEF CTX KAN NAL	AMP TET NAL SXT	AMP STR NAL	STR TET NAL	TET NAL CHL	STR NAL CIP	AMP STR	STR TET	TET CHL	AMP NAL	NAL	AMP	SXT
59	76.0%	-	-	1.0%	1.0%	15.6%	-	1.0%	1.0%	4.2%	-	-	-	-
59:2.1	27.6%	-	-	-	-	3.4%	-	-	-	-	-	69.0%	-	-
59:3.6:2.1	81.5%	-	-	-	-	-	-	-	-	-	-	18.5%	-	-
59:3.9:2.1	37.0%	-	-	-	-	-	-	-	-	-	-	63.0%	-	-
59:39:2.1	80.0%	3.3%	-	-	-	-	-	-	-	-	-	13.4%	-	3.3%
59:4.5:2.1	29.1%	-	-	-	-	-	-	-	-	1.8%	-	69.1%	-	-
59:45	39.3%	-	-	-	-	-	3.6%	-	-	-	-	57.1%	-	-
59:45:2.1	63.6%	-	9.1%	-	-	-	-	-	-	-	9.1%	-	18.2%	-
59:45:3.4	35.7%	-	-	-	-	-	-	-	-	-	3.6%	53.6%	7.1%	-
59:6.7	27.3%	-	-	-	-	-	-	-	-	4.5%	-	54.6%	-	-
Total (n = 345)	53.3%	0.3%	2.8%	0.3%	0.3%	4.6%	0.3%	0.3%	0.3%	1.7%	0.6%	35.4%	1.2%	0.3%

Antibiotics used: Ampicillin 10 μg (AMP), cephalexin 30 μg (CEF), cefuroxime 30 μg (CXM), cefotaxime 30 μg (CTX), cefepime 30 μg (FEP), streptomycin 10 μg (STR), kanamycin 30 μg (KAN), gentamicin 30 μg (GEN), amikacin 30 μg (AMK), tetracycline 30 μg (TET), nalidixic acid 30 μg (NAL), ciprofloxacin 5 μg (CIP), imipenem 10 μg (IMI), chloramphenicol 30 μg (CHL), and trimethoprim 1.25 μg/sulfamethoxazole 23.75 μg (SXT).

**Table 2:** Antibiotic susceptibility of *S.* Enteritidis strains (n = 345) isolated from different ecological niches in regions of Siberia and the Far East of Russia.

*S.* Enteritidis Strains Isolated from Different Ecological Niches (n = 345)	Susceptibility	Number of Strains (%)														
		AMP	CEF	CXM	CTX	FEP	STR	KAN	GEN	AMK	TET	NAL	CIP	IMI	CHL	SXT
Sporadic cases (n = 255)	R ^1^	2.7	0.4		0.4		1.6	0.7	0.4		5.9	53.7	0.4		5.0	1.2
	I ^2^		0.4	5.9	0.4	0.4	11.4	51.8	14.1	9.0	1.6	2.0	8.2			0.4
	S ^3^	97.3	99.2	94.1	99.2	99.6	87,1	47.4	85.5	91.0	92.5	44.3	91.4	100	95.0	98.4
Outbreak cases (n = 41)	R						2.4				14.6	61.0			12.2	
	I			7.3			4.9	34.1	2.4			2.4				
	S	100	100	92.7	100	100	92.7	65.9	97.6	100	85.4	36.7	100	100	87.8	100
Food (n = 46)	R	2.2									4.3	52.2			4.3	
	I			8.7			2.2	32.6	8.7	2.2	2.2	4.3	10.9			
	S	97.8	100	91.3	100	100	97.8	67.4	91.3	97.8	91.3	43.5	98.1	100	95.6	100
Environment (n = 3)	R										66.7	66.7			66.7	33.3
	I							33.3								
	S	100	100	100	100	100	100	66.7	100	100	33.3	33.3	100	100	33.3	66.7

^1^ R—resistant, ^2^ I—Intermediate, ^3^ S—Susceptible. Antibiotics used: Ampicillin 10 μg (AMP), cephalexin 30 μg (CEF), cefuroxime 30 μg (CXM), cefotaxime 30 μg (CTX), cefepime 30 μg (FEP), streptomycin 10 μg (STR), kanamycin 30 μg (KAN), gentamicin 30 μg (GEN), amikacin 30 μg (AMK), tetracycline 30 μg (TET), nalidixic acid 30 μg (NAL), ciprofloxacin 5 μg (CIP), imipenem 10 μg (IMI), chloramphenicol 30 μg (CHL), and trimethoprim 1.25 μg/sulfamethoxazole 23.75 μg (SXT).

## Data Availability

No new data were created or analyzed in this study. Data sharing is not applicable to this article.
